# Influenza Virus-Like Particles Containing M2 Induce Broadly Cross Protective Immunity

**DOI:** 10.1371/journal.pone.0014538

**Published:** 2011-01-18

**Authors:** Jae-Min Song, Bao-Zhong Wang, Kyoung-Mi Park, Nico Van Rooijen, Fu-Shi Quan, Min-Chul Kim, Hyun-Tak Jin, Andrew Pekosz, Richard W. Compans, Sang-Moo Kang

**Affiliations:** 1 Department of Microbiology and Immunology and Emory Vaccine Center, Emory University School of Medicine, Atlanta, Georgia, United States of America; 2 Department of Molecular Cell Biology, Vrije Universiteit Medisch Centrum, Amsterdam, The Netherlands; 3 Department of Molecular Microbiology and Immunology, Johns Hopkins University, Baltimore, Maryland, United States of America; IGMM CNRS 5535, France

## Abstract

**Background:**

Current influenza vaccines based on the hemagglutinin protein are strain specific and do not provide good protection against drifted viruses or emergence of new pandemic strains. An influenza vaccine that can confer cross-protection against antigenically different influenza A strains is highly desirable for improving public health.

**Methodology/Principal Findings:**

To develop a cross protective vaccine, we generated influenza virus-like particles containing the highly conserved M2 protein in a membrane-anchored form (M2 VLPs), and investigated their immunogenicity and breadth of cross protection. Immunization of mice with M2 VLPs induced anti-M2 antibodies binding to virions of various strains, M2 specific T cell responses, and conferred long-lasting cross protection against heterologous and heterosubtypic influenza viruses. M2 immune sera were found to play an important role in providing cross protection against heterosubtypic virus and an antigenically distinct 2009 pandemic H1N1 virus, and depletion of dendritic and macrophage cells abolished this cross protection, providing new insight into cross-protective immune mechanisms.

**Conclusions/Significance:**

These results suggest that presenting M2 on VLPs in a membrane-anchored form is a promising approach for developing broadly cross protective influenza vaccines.

## Introduction

Vaccination is the most effective measure to control influenza. Current influenza vaccines are based primarily on antibody responses against the viral glycoprotein hemagglutinin (HA). HA-specific antibodies neutralize viral infectivity and protect against infection, which is the principle protective correlate of available human influenza vaccines. A limitation of current vaccines is that the major vaccine targets, the antigenic regions of HA, are highly susceptible to continuous mutation in circulating epidemic virus strains [1], [2]. The high mutation rate of the viral genome and the selection of mutants in the human host population result in antigenic drift from the previous circulating strains [3]. In some cases, novel pandemic strains can occur by reassortment of genes between animal and human viruses [4]. The emergence of the 2009 pandemic H1N1 virus is a good example of the generation of a new strain by triple reassortments with distinct antigenic properties different from the circulating seasonal influenza viruses [5], [6]. While antibodies to HA provide potent virus strain-specific protection, the vaccine formulations need to be evaluated on a yearly basis to match the current circulating strains. The development of a vaccine that can confer cross protection against different influenza variants and subtypes is highly desirable, and may limit the need for annual vaccination.

In contrast to HA, the influenza A M2 protein has a highly conserved extracellular domain of 23 amino acids (M2e). However, due to its small size and low immunogenicity, previous studies have focused on M2e peptide fusion constructs using a variety of carrier molecules: hepatitis B virus core [7]–[9], human papilloma virus L protein [10], keyhole limpet hemocyanin [11], bacterial outer membrane complex [8], [12], liposome [13], and flagellin [14]. M2 vaccines based on M2e fusion carriers or DNA – recombinant vector combination could provide cross protection against lethal infection with different strains [8], [11], [13], [15]. These studies suggested that M2e antibody played an important role in providing protection. However, previous studies on M2e conjugate vaccines used potent adjuvants such as cholera toxins or heat labile endotoxins' derivatives, saponin QS21, Freund's adjuvants, or bacterial protein conjugates [8], [9], . Such adjuvants that nonspecifically elicit host responses including inflammation are potentially adverse and unwarranted in developing a widely applicable prophylactic influenza vaccine. More over, the longevity and breadth of cross protection mediated by M2 immunity remain largely unknown.

Influenza virus-like particles (VLPs) containing HA and/or neuraminidase (NA) on their surfaces in a membrane-anchored form have been demonstrated to provide effective protection suggesting a promising vaccine modality (reviewed in [18]). The M2 protein is expressed as a tetrameric protein in a membrane anchored form [19], [20]. Therefore, it was likely that M2 would be incorporated into VLPs in a native conformation during the budding process on the cell surface. In this study, we investigated the generation of VLPs containing the wild type M2 protein as well as their immunogenicity, long-term cross-protective efficacy, and the breadth of cross protection against heterologous and heterosubtypic influenza strains even with a different M2e sequence. In addition, the potential protective mechanisms of immune responses to the M2 antigen are investigated and discussed.

## Results

### Preparation of VLPs containing the A/WSN M2 protein

**Figure 1:**
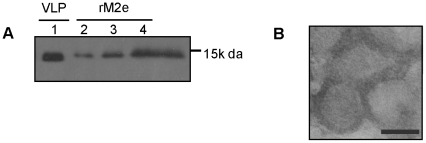
Characterization of influenza M2 VLP. (A) Western blotting of M2 VLP and recombinant M2e protein. M2 VLP (Lane 1; 0.5ug total protein) and recombinant M2e protein (Lane 2, 3, 4; 15, 30 60 ng respectively) were loaded and detected by western blotting using mouse anti-M2e monoclonal antibody (14C2). Amount of M2e protein incorporated in M2 VLP was calculated by spot densitometry analysis using serial diluted rM2e protein as a standard. (B) Negative staining electron microscopy of influenza M2 VLP (bar = 100 nm).

To investigate the role of M2 in inducing cross protection against heterologous viruses, we produced influenza VLPs containing the wild type M2 protein derived from influenza A/WSN/33 virus (H1N1) (M2 VLPs). M2 VLPs were produced in insect cells coinfected with recombinant baculoviruses (rBVs) expressing M1 and M2, purified using sucrose gradient ultracentrifugation, and characterized by western blot using anti-M2 monoclonal antibody 14C2 [21]. The amount Of M2 protein incorporated into VLPs was estimated to be approximately 1% of the total protein (Fig. 1A). M2 VLPs produced in insect cells were examined by transmission electron microscopy after negative staining of VLPs (Fig. 1B). Spherical particles similar to the size of virus were observed. Control M1 VLPs showed similar morphology as M2 VLPs (not shown).

### M2 VLPs induce M2-specific and broadly cross-reactive antibody responses

**Figure 2:**
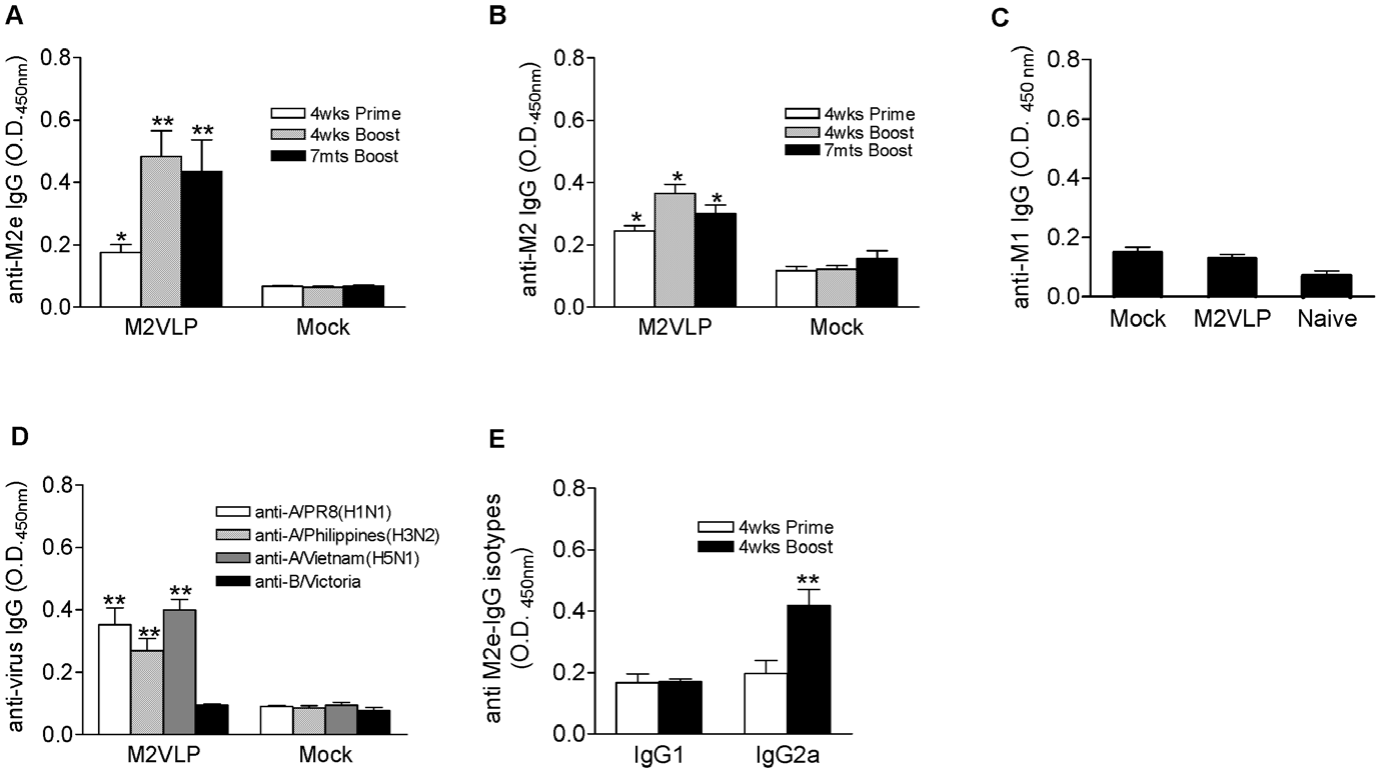
Antibody responses after M2 VLP vaccination. Groups of mice (n = 9) were intranasally immunized with 20 µg of M2 VLPs (M2VLP) or M1 only VLPs without M2 (Mock) two times at weeks 0 and 4. Serum samples were taken 4 weeks after priming (4wks Prime) and at 4 weeks and 7 months after boost immunization (4wks Boost, 7mts Boost). The IgG antibody levels against M2e peptide (A), M2 expressed on cell surfaces (B), M1 peptides (C), and virus coated plates (D) were determined by ELISA and presented as optical density values read at 450nm (100× diluted sera). IgG1 and IgG2a isotype titers (E) against M2e peptide were determined by ELISA. Values mean average±S.D. of 1∶100 diluted serum samples. The asterisk indicates a significant difference between M2VLP and Mock groups, ** p<0.01; * p<0.05.

To determine the immunogenicity of influenza VLPs containing M2, a group of mice (6 BALB/c mice per group) was immunized intranasally with VLPs containing M2 (20 µg total proteins) once or twice at weeks 0 and 4. Levels of M2-specific IgG antibodies were determined at 4 weeks after priming or at 4 weeks and 7 months after boost immunizations by ELISA using the M2 ectodomain peptide as a coating antigen (Fig. 2A). M2 specific antibodies were detected in the M2 VLP immunized group (M2VLP) at significant levels after priming with M2 VLPs. A control group of mice that was immunized with M1 VLPs not containing M2 did not show an M2 specific antibody response (Mock). After boost immunization, levels of antibodies specific to M2 were increased by over 2 fold (Fig. 2A). M2 immune sera showed binding reactivity to M2 expressed on the cell surfaces (Fig. 2B), indicating that M2 antibodies recognize the native form of M2. This result is consistent with those observed by immunization with the tetrameric ectodomain of M2 GCN4 conjugate vaccines [22]. Low but detectable levels of antibodies to an M1 peptide pool antigen were observed in the M1 only VLP group (Fig. 2C), which were similar to the M2 VLP group. Therefore, these results indicate that vaccination with M2 VLPs can induce M2 specific antibody responses that are long lived for over 8 months.

Since M2 is a protein which is conserved among various influenza A strains, we tested whether M2 VLP immune sera would be cross reactive with different influenza A virus subtypes. Immune sera collected from mice boosted with M2 VLPs showed significant levels of antibodies cross reactivities to influenza H1N1 (A/PR/8/34) as well as the heterosubtypic H3N2 A/Philippines/82 virus (Fig. 2D). Importantly, M2 VLP immune sera also showed significant levels of cross reactivity to an H5N1 virus (A/Vietnam/1203/04) (Fig. 2D) and a 2009 H1N1 virus (A/California/4/2009) (not shown) which have an M2 protein with different amino acid sequences compared to the M2 protein (A/WSN/33) used for vaccination (Table 1). In contrast, antibodies cross-reactive to influenza B/Victoria were not observed in the M2 VLP immune sera (Fig. 2D), indicating that M2 VLP vaccination induce cross-reactive antibodies to the influenza A but not B type virus. To determine the antigenic specificity, purified hemagglutinin (HA) derived from A/PR/8/34 or A/Vietnam/1203/2004 was used as an ELISA coating antigen. We found that there was no significant reactivity to HA in M2 VLP immune sera (data not shown), indicating that cross reactivity to different influenza viruses is likely to be specific to M2 independent of HA subtype. When we determined IgG1 and IgG2a isotype antibodies specific to M2 peptide antigen, IgG2a was found to be predominant after boost immunization (Fig. 2E). Therefore, these results suggest that M2 VLPs are immunogenic and capable of inducing antibodies cross-reactive to influenza A virions independent of HA subtype and in the absence of adjuvant.

**Table 1:** M2e amino acid sequence of influenza A viruses.

Viral strains	M2e amino acid sequence		
A/WSN/33 (H1N1)	MSLLTEVETP	IRNEWGCRCN	DSSD
A/Puerto Rico/8/34 (H1N1)^1^	-------------------	--------------------	G------
A/Philippines/82 (H3N2)	-------------------	---------------------	---------
A/California/4/09 (2009 H1N1)^2^	-------------------	T--S-----E--------S	---------
A/Vietnam/1203/04 (H5N1)^3^	-------------------	T-------- E------- S	----------

Genebank Accession numbers:

1 1NP_040979.2;

2 FJ969513.1;

3 ABF01919.1.

### M2 VLPs provide protection against both H1N1 and H3N2 viruses

**Figure 3:**
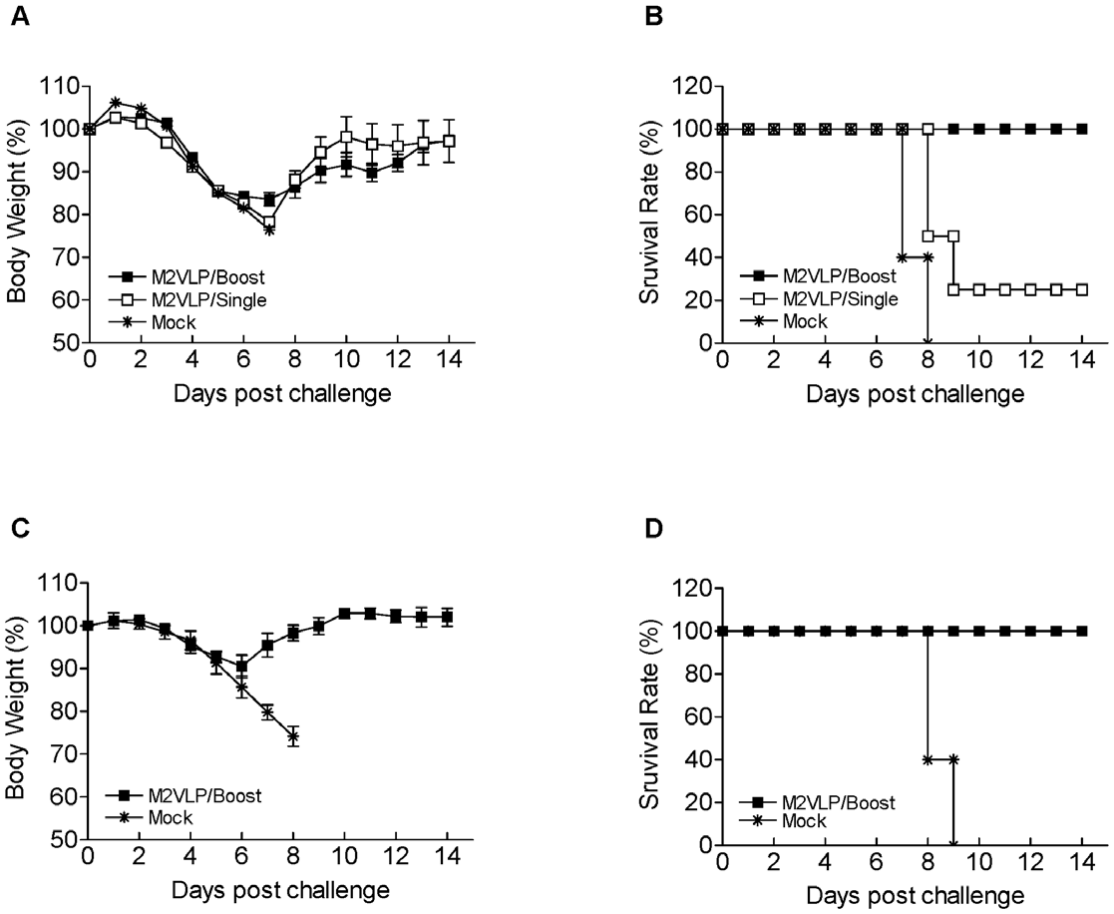
Protection against heterologous lethal challenge. (A–B) Groups of vaccinated mice and a mock control were intranasally challenged with a lethal dose (3× LD_50_) of A/PR/8/34 (H1N1) influenza virus at 4 weeks after prime (n = 4) or boost (n = 9) vaccination. Body weight changes (A) and survival rates (B) were recorded for 14 days. M2VLP/single, one time immunization with M2 VLPs, M2VLP/boost, prime-boost immunizations with M2 VLPs, Mock: prime-boost immunizations with M1 only VLPs without M2. (C–D) A lethal dose (3× LD_50_) of A/Philippines/82 (H3N2) influenza virus was used to challenge the mice vaccinated with two doses of M2 VLPs. Body weight changes (C) and survival rates (D) were recorded daily (n = 5 mice out of 9). Similar body weight changes and 100% protection were reproducible in duplicate experiments.

To determine the ability of M2 VLPs to confer cross protection against heterologous lethal challenge infection, groups of mice intranasally immunized with M1/M2 VLPs (M2 VLPs) or M1 VLPs without M2 (Mock) were challenged with a lethal dose (3 LD_50_) of heterologous H1N1 A/PR/8/34 virus or heterosubtypic H3N2 A/Philippines/82 virus at 4 weeks after vaccination (Fig. 3). The body weight changes and survival rates were monitored following challenge infection. All mock controls lost over 25% in body weight and had to be euthanized (Fig. 3A). The mice that received a single dose of M2 VLPs showed approximately 20% body weight loss resulting in a survival rate of 25%. In contrast, the mice that received prime boost immunizations with M2 VLPs were 100% protected against lethal infection with A/PR8 virus (Figs. 3A, B). These mice showed a loss of approximately 18% in body weight at day 7 post challenge and then recovered to the normal body weight. Similar to lethal challenge infection with A/PR8 virus, the prime-boost immunized mice were also completely protected against lethal challenge with A/Philippines virus and showed a transient loss of 10% in body weight (Figs. 3C, D). These results demonstrate that M2 VLP vaccination can provide protection against lethal infection with either H1N1 A/PR8 or H3N2 A/Philippines virus despite some accompanying morbidity as shown by body weight loss.

### M2 VLPs induce mucosal IgA antibodies and lower lung viral titers

**Figure 4:**
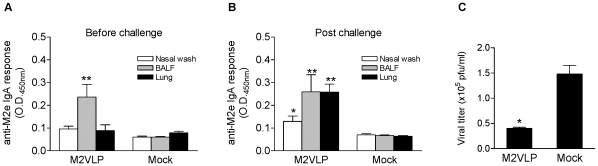
M2 VLP vaccination induces mucosal IgA antibodies and lowers lung viral replication. Nasal wash, BALF, and lung samples were collected from individual mice before challenge (A) and at day 4 post challenge (B) with A/Philippines/82 (H3N2) virus (n = 6) 4 weeks post boost vaccination. Nasal wash (2× diluted), BALF (2× diluted), and lung homogenates (4× diluted) were used for determination of IgA antibody responses specific to M2 peptide (A–B). Lung virus titers (C) were determined by using a plaque assay. The asterisk indicates a significant difference between M2VLP and Mock groups, * p<0.05, ** p<0.01.

Mucosal immunity is important for conferring cross protection. We therefore determined M2 specific IgA antibody responses in various mucosal tissues after vaccination (Fig. 4A). Significant levels of IgA antibody responses were observed in bronchoalveolar lavage fluid (BALF) but not in nasal wash and lung samples (Fig. 4A). Induction of mucosal IgA antibodies after vaccination is consistent with results reported using intranasal vaccination with M2 DNA and/or recombinant adenovirus vector vaccines [23], [24]. Interestingly, a rapid increase in lung IgA antibodies specific to M2 peptide was observed in the M2 VLP-immunized group but not in the mock control group at day 4 after challenge compared to that before challenge (Fig. 4B). Also, moderate levels of IgA antibody responses were detected in nasal wash after challenge (Fig. 4B). To better assess heterosubtypic cross protective efficacy against A/Philippines/82 (H3N2), lung viral titers were determined at day 4 after challenge. The group of mice intranasally immunized with M2 VLPs, which showed 100% protection against A/Philippines/82, had 4 fold lower lung viral titers compared to that in the mock control group (Fig. 4C). Therefore, it is likely that M2 specific immune responses including IgA antibodies in BALF and lungs can effectively contribute to controlling heterosubtypic virus replication.

### M2 VLPs induce M2-specific T cell responses

**Figure 5:**
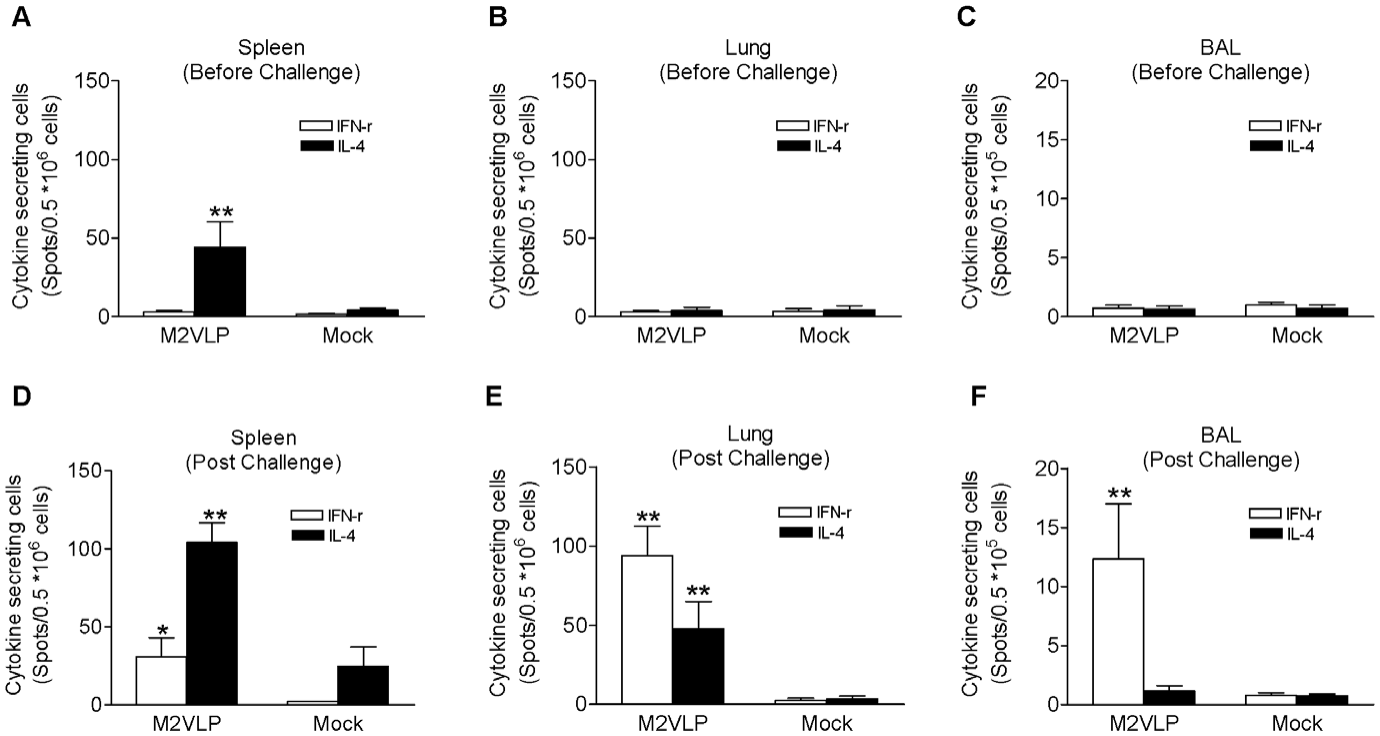
Cellular immune responses. The cellular immune responses were assessed with splenocytes isolated from mice 4 weeks boost immunization with M2 VLPs or M1 only VLPs without M2 (Mock) before challenge (A–C) and at 4 day post challenge (D–F) with A/Philippines/82 virus (n = 6). Cells from spleen (A, D), lung (B, E), and BAL (C, F) were stimulated with M2 peptides for 2 days and cytokine forming cell spots were determined by ELISPOT assay. The asterisk indicates a significant difference between M2VLP and Mock groups, * p<0.05, ** p<0.01.

T cell responses are known to contribute to broadening cross protective immunity. After *in vitro* stimulation of cells with the M2 specific peptide, cytokine producing cell spots were measured as an indicator of T cell responses (Fig. 5). Intranasal vaccination with M2 VLPs induced IL-4 secreting cells in spleens but not in lung and BAL samples (Fig. 5A–5C). IFN-γ secreting cells were not detected after vaccination. To determine recall immune responses of M2-specific T cells, spleen cells were collected at day 4 after challenge from mice 4 weeks post vaccination with M2 VLPs. Significant levels of IFN-γ secreting cells were observed in spleens although their levels were lower than those of IL-4 secreting cells (Fig. 5D). Interestingly, spot numbers of IFN-γ secreting cells were significantly higher in lungs and BAL samples after challenge than those of IL-4 (Fig. 5E–5F). The mock control group immunized with VLPs without M2 showed background levels similar to media only without M2 peptide stimulation. These results provide evidence that M2 VLP immunization can induce M2 specific IL-4 secreting T cell responses in spleens and IFN-γ secreting recall T cell responses in mucosal tissues.

### M2 VLP vaccination induces long-lasting protective immunity

**Figure 6:**
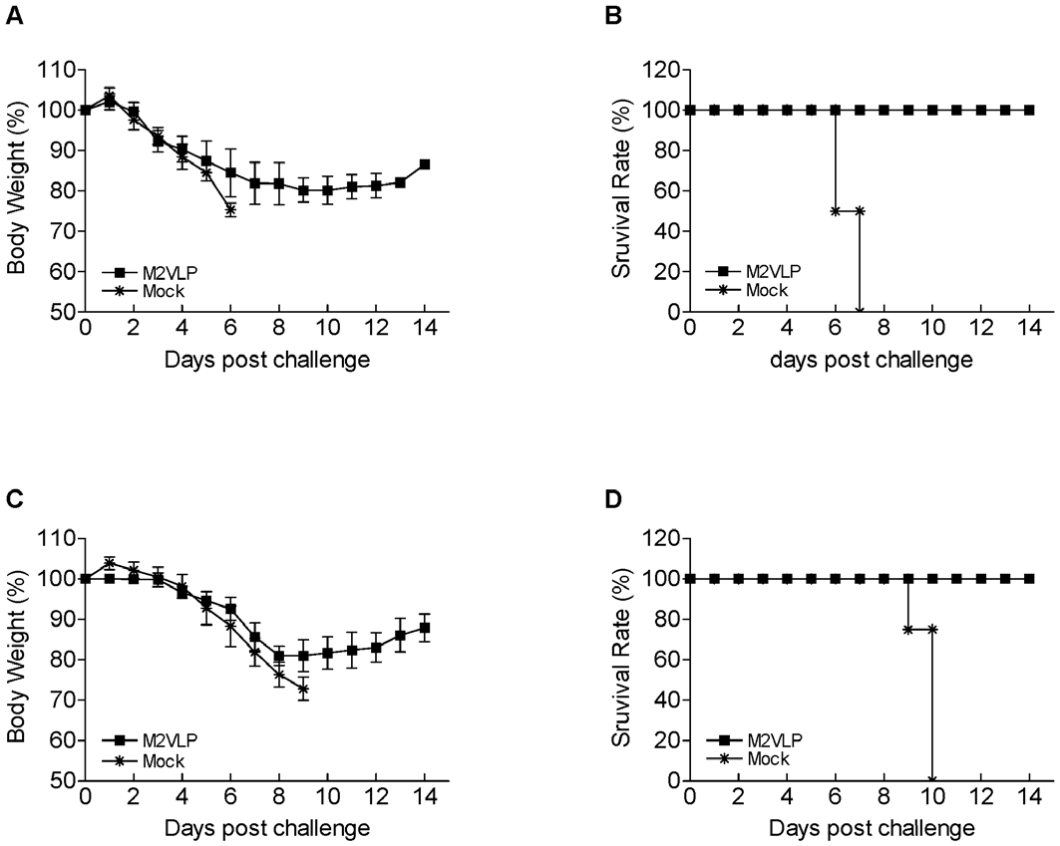
Long-term protection against heterologous lethal challenge by M2 VLP vaccination. (A–B) At six months after prime-boost immunizations with M2 VLPs, mice (n = 5) were challenged with a lethal dose (3× LD_50_) of A/PR/8/34 (H1N1) influenza virus. A) Body weight changes, B) Survival rates. (C–D) Mice (n = 5) that were intranasally immunized with M2 VLPs 7 months earlier were challenged with A/Philippines/82 (H3N2) virus. Body weight changes (C) and survival rates (D) are shown.

The longevity of protective immunity after vaccination with conjugate M2e vaccines has not been previously reported. To determine the longevity of protective immunity induced by M2 VLP vaccination, groups of mice that were intranasally immunized with M2 VLPs were challenged with A/PR/8/34 virus (Figs. 6A, B) or A/Philippines/82 (Figs. 6C, D) at 6 and 7 months after boost vaccination respectively. Mice that were intranasally immunized with M2 VLPs (20 µg total protein) using a prime boost regimen were 100% protected against lethal challenge infection with A/PR/8/34. Also, a group of mice that was intranasally immunized twice with a lower dose of M2 VLPs (10 µg) was 100% protected against a lethal challenge with H3N2 A/Philippines virus These results show that M2 VLP vaccines can confer long-lasting protection against lethal infection.

### M2 VLP immune serum contributes to protective immunity

**Figure 7:**
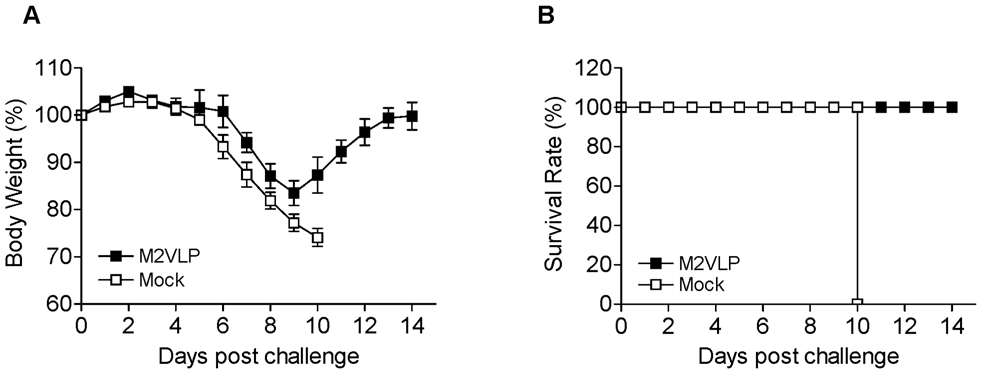
Protective efficacy against influenza A H3N2 virus of M2 VLP immune sera. Immune sera collected from M2 VLP vaccinated mice at 4 weeks after boost vaccination were incubated with a lethal dose of A/Philippines/82 (H3N2) influenza virus at room temperature for 30 min. Groups of mice (n = 4) were intranasally challenged with a lethal infectious dose mixed with M2 immune sera (M2VLP) or Mock sera. Body weight (A) and survival rate (B) were monitored for 14 days. 100% protection and similar body weight changes were obtained from sera of the M2VLP group in duplicate experiments.

To better understand the protective role of anti-M2 immune sera, we tested the capability of M2 immune sera to provide protection in naïve mice when infected with a lethal challenge. Mixtures of a lethal dose of A/Philippines/82 virus (H3N2) and M2 immune or naïve sera were used to intranasally infect naïve mice. This method is a sensitive and previously well established assay to assess the protective role of polyclonal immune sera [25]–[27]. Naïve mice that were infected with a mixture of virus and mock immune sera showed severe body weight loss reaching to below 75% of original weights by day 10 post-infection and all had to be euthanized (Fig. 7). In contrast, M2 immune sera provided complete protection to naïve mice that were infected with a lethal dose of A/Philippines/82 virus. Therefore, these results suggest that anti-M2 immune sera play an important role in providing heterosubtypic cross protection.

**Figure 8:**
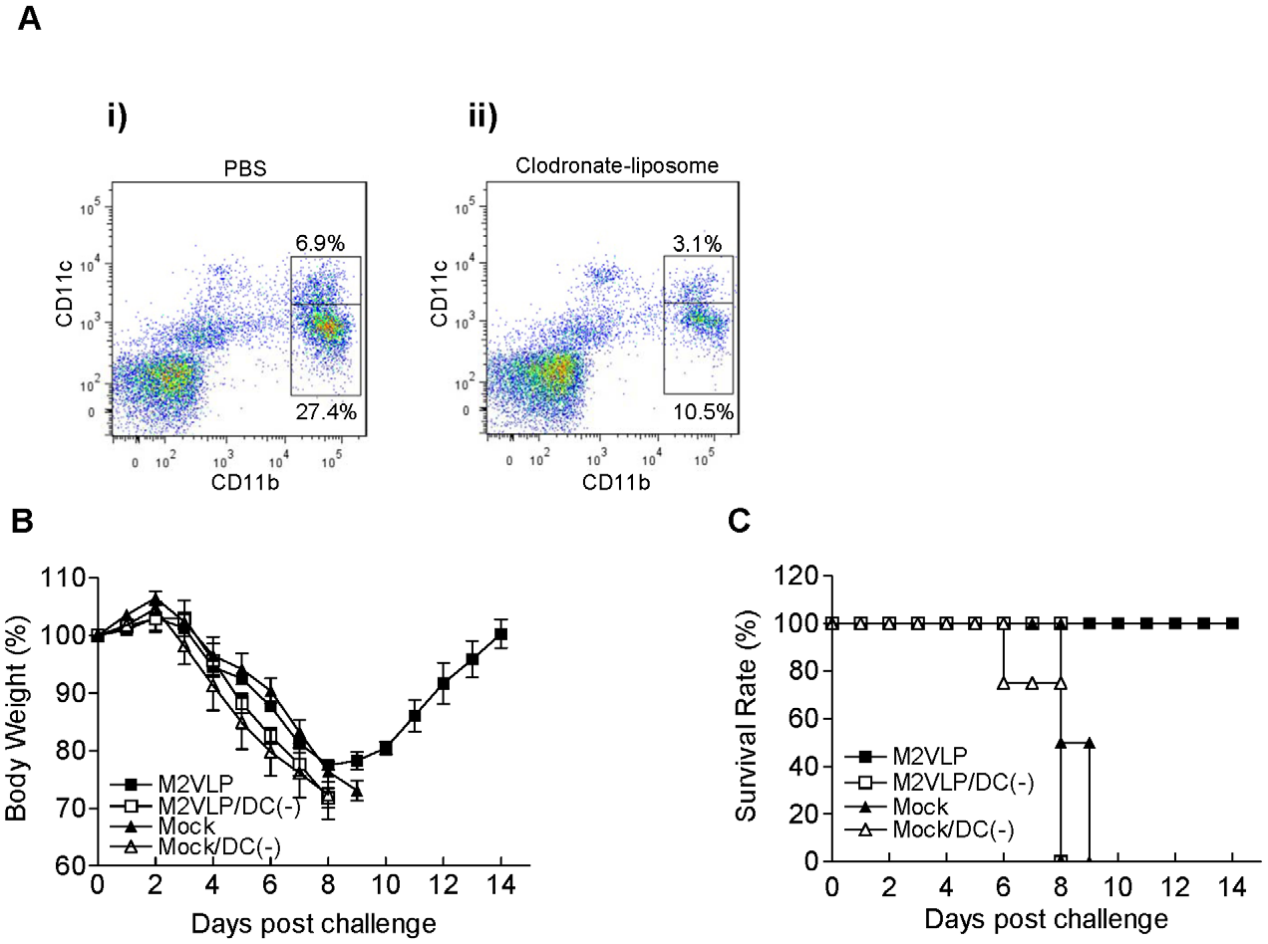
Effects of clodronate-liposomes on protective efficacy against 2009 H1N1 virus by M2 immune sera. (A) A representative flow cytometry profile of DC and macrophage cell gates. Naive mice were intranasally treated with PBS (i) or clodronate-liposome (ii), and DC cells (CD11b^+^CD11c^+^) and macrophage cells (CD11b^+^CD11c^−^) in lungs were analyzed by flow cytometry. (B–C) Roles of DC/macrophage cells in the cross protection mediated by M2 immune sera. (B) Body weight changes against A/Califonia/04/2009. (C) Survival rates against A/Califonia/04/2009. To deplete dendritic cells (DC) and alveolar macrophages, groups of naïve mice (n = 4) were intranasally instilled with clodronate-liposomes 4 hrs prior to lethal infection. A lethal dose of A/Califonia/04/2009 (H1N1) influenza virus was incubated with sera collected from M2 VLP vaccinated mice at 4 weeks after boost immunization and used to infect naïve mice with or without clodronate-liposome pretreatment. M2VLP: M2VLP immune sera in mice without clodronate-liposome; M2VLP/DC(-):M2VLP immune sera in mice with clodronate-liposome, Mock: Mock immune sera in mice without clodronate-liposome; Mock/DC(-):Mock immune sera in mice with clodronate-liposome.

Next, we studied the potential roles of lung airway dendritic and macrophage cells in conferring anti-M2 antibody-mediated cross protection against the antigenically distinct 2009 pandemic H1N1 virus (Fig. 8). Previous studies demonstrated the selective depletion of lung dendritic and macrophage cells by intranasal or intratracheal administration with clodronate-liposomes [28], [29]. Similarly, we sought to selectively deplete lung dendritic and macrophage cells by intranasal administration of clodronate-liposomes. As shown in Fig. 8A, CD11c^+^ DC and CD11b^+^ macrophage cells were found to be depleted by 55% and 62% respectively after clodronate-liposome treatment, which is consistent with the depletion efficiency reported in a previous study [29]. Naïve mice with or without clodronate-liposome treatment were infected with a lethal dose of infectious virus mixed with M2 immune sera (Fig. 8B, 8C). M2 VLP immune sera could also transfer cross protection to naïve mice from a lethal infection with a 2009 H1N1 virus (Fig. 8), as was seen with H3N2 A/Philippines/82 virus (Fig. 7). Regardless of treatment with clodronate-liposomes (Mock, Mock/DC(-)), mock immune sera did not provide any protection against the 2009 pandemic H1N1 virus. Importantly, naïve mice that received clodronate-liposome treatment were not protected against the 2009 H1N1 virus mixed with M2 VLP immune sera, and all mice in this group died. These results indicate that dendritic and macrophage cells might be important for conferring M2 immune serum-mediated cross protection.

## Discussion

M2 vaccines in a membrane-anchored form on VLPs have not been previously tested for their cross protective efficacy. Results in this study demonstrate that M2 VLP vaccination can induce M2 specific antibody responses cross reactive to heterologous viruses, T cell responses, and long-lasting protective immunity against lethal challenges with heterologous or heterosubtypic viruses. Influenza M2 vaccine approaches based on VLPs are desirable since these VLPs were found to be immunogenic in the absence of adjuvant. In contrast, conjugate-based M2 vaccines required the use of adjuvants that would be problematic for human use [8], [9], [12], [14], [16], [17]. Therefore, developing influenza M2 vaccines based on VLPs is significant since VLPs containing M2 can be easily produced, and are safe and practical for public health use.

Previous studies have focused on chemical or genetic fusion constructs of the M2 extracellular peptide domain (M2e), which were shown to provide partial or complete protection against lethal infection in animal models (reviewed in [30]). M2 immunity and protection were reported by vaccination of animals with M2e peptide in chemical or genetic conjugates (carrier molecules or virus particles) [7]–[9], [12], [14], [16], [17], [22], [31], or DNA vaccines and/or combination of DNA and recombinant or live influenza vaccines [11], [23], [24], [32]. These conjugate or genetic M2e vaccines even together with potent adjuvants were not completely protective since vaccinated animals showed disease symptoms visible by weight loss. Chemical or genetic conjugation of M2e would not represent M2 in its tetrameric membrane-anchored native form. As a vaccine antigen, use of the wild type M2 protein would be advantageous since it is likely to present M2 in a native conformation. In the present study, influenza M2 VLPs were produced by a budding process in insect cells expressing influenza M1 and M2. Consistent with the results in previous studies reporting weak protective immunity to M2, vaccination with M2 VLPs in the absence of adjuvant did not prevent body weight losses in protected mice. Approaches to increase the immunogenicity of M2 VLPs include chimeric or multi-components VLPs incorporating HA or molecular adjuvants into M2 VLPs. We have demonstrated enhanced immunogenicity of chimeric VLPs containing granulocyte-macrophage colony stimulating factor [33], which could further improve cross protective efficacy possibly preventing weight loss.

Our study demonstrated that M2 VLP vaccination induces antibodies binding to the M2 extracellular peptide, the native form of M2 expressed on cell surfaces, as well as to purified virions regardless of HA subtype. It is likely that M2 specific antibodies induced by M2 VLP vaccination recognize the native form of M2 on virions. It was reported that vaccination with recombinant M2e fused to the oligomerization domain of GCN4 induced antibodies binding to cell surfaces expressing M2 [22]. Although anti-M2 antibodies may not directly neutralize the virus [34], influenza viruses bound to M2 antibodies might be preferably recognized and removed by opsonophagocytosis by macrophages. In support of this idea, it was demonstrated that M2 monoclonal antibodies which preferentially bind to M2 multimeric forms but not the monomeric form were protective, and that this was independent of natural killer cell mediated effector functions [35]. In a previous study, M2 antibodies induced by M2e conjugate vaccination did not efficiently bind to the free virus particles, and natural killer cell-dependent elimination of infected cells was shown to contribute to the relatively weak protection observed [34], suggesting an alternative mechanism for M2 antibody mediated protection.

IgG2a antibody is an isotype known to interact efficiently with complement and Fc receptors [36]–[39]. We found that IgG2a was the predominant isotype induced by M2 VLP vaccination and that mice immunized with M2 VLPs showed reduced lung viral titers. Huber et al. demonstrated that non-neutralizing anti-influenza humoral immunity was dependent on opsonophagocytosis of influenza virions by macrophages [36], [40]–[42]. Therefore, we suggest that induction of virus-binding IgG2a antibodies by M2 VLP vaccination contributes to viral clearance, possibly via opsonizing virus by macrophages and dendritic cells. We found that M2 VLP immune sera were able to confer protection against lethal infection in naïve mice, indicating that anti-M2 antibodies play an important role in providing protection against lethal infection. Furthermore, we obtained evidence that this protection by M2 immune sera might be mediated by dendritic and macrophage cells as shown by depletion experiments using clodronate-liposomes. A possible explanation is that anti-M2 antibodies may by itself be too weak to protect the mice and may just contribute to protection. At the same time, dendritic cells and macrophages which are not by themselves sufficient for protection may contribute to innate and early adaptive immune responses triggered by challenge infection and enhancing the virus uptake of antibody bound particles via antigen presenting cells. Therefore, the combination of those responses and the anti-M2 antibodies may protect the mice. In support of this hypothesis, we observed rapid increases in levels of lung IgA antibodies and IFN-γ secreting cell responses at an early time post challenge. Further studies are ongoing to better understand the M2-immune mediated protection mechanism.

Our results demonstrated that M2 VLP vaccination was able to induce long-lasting M2e specific antibodies and to provide protection against lethal challenge even at 7 months after vaccination. Thus, it is possible that long-lived M2-specific antibody responses might contribute to conferring long-term protective immunity since antibody-mediated immunity is usually long-lived and most successful antiviral vaccines are based on the induction of protective antibody responses [43]–[45]. Also, in a previous study using NP/M2 DNA prime - NP/M2 recombinant adenovirus vector boost regimen, intranasal immunization induced long-lived NP/M2 specific IgG and IgA antibodies in sera and mucosal sites [24]. In addition, M2 immunity might be affected depending on the genetic background of mouse species [46], suggesting that M2 alone immunity observed in BALB/c mice might not be well translated to heterogeneous human population. The influenza M1 component in VLPs and/or combination vaccination with conserved NP might provide better coverage of human leukocyte antigen haplotypes in the genetically diverse human population [24], [47]. Alternatively, incorporating immunostimulatory molecules into VLPs might be an approach for inducing M2 immunity independent of CD4 T cell help [48]. Therefore, further studies on M2 vaccination are needed to improve cross protective immunity and to prevent morbidity as shown by body weight loss.

In summary, we observed that M2 VLP vaccination induced in the absence of adjuvant protective immunity against a H1N1 virus (A/PR/8/34) as well as an H3N2 subtype virus (A/Philippines/82). Also, M2 VLP immune sera can provide protection to naïve mice against the 2009 pandemic H1N1 virus (A/California/4/2009) that contains differences in the M2 protein sequence (Table 1). Cross protection was observed up to 7 months post vaccination, suggesting that M2 VLP based protective immunity is long-lasting. Therefore, we believe that M2 VLP vaccines would be safe, convenient, and practical for public health use. Further studies are needed to develop improved vaccines based on influenza M2 VLPs, a highly conserved target which can be applied as a universal vaccine against influenza A viruses.

## Materials and Methods

### Cells, Viruses, and Reagents

*Spodoptera frugiperda* sf9 insect cells (ATCC, CRL-1711) were maintained in SF900-II serum free medium (Invitrogen, Carlsbad, CA) at 27°C and used for production of recombinant baculoviruses (rBVs) and VLPs. Madin-Darby canine kidney (MDCK) cells used for viral titration were purchased from ATCC and cultured in Dulbecco's Modification of Eagle's Medium (DMEM) with 10% fetal bovine serum [26]. Influenza A viruses, A/California/4/2009 (2009 pandemic H1N1 virus) kindly provided by Dr. Richard Webby, mouse adapted A/Philippines/2/1982 (H3N2) and A/PR/8/34 (H1N1) generously provided by Dr. Huan Nguyen, and influenza B virus (B/Victoria/2/87, ATCC) were propagated in the allantoic cavity of 11 day-old embryonated chicken eggs for 48 hrs at 37°C. Harvested allantoic fluid was clarified by centrifugation (3000 rpm, 30min) and kept at −80°C. Inactivated A/VietNam/1203/04 (H5N1) influenza virus and purified H5 HA soluble protein were obtained from the NIH Biodefense and Emerging Infections Research Resources Repository (NIAID, NIH). A recombinant H1 HA protein derived from the A/PR8 strain was expressed using the baculovirus expression system and purified using a His-Tag affinity column. Influenza virus M2e (17 amino acids 2 to 18, N-SLLTEVETPIRNEWGCR) was synthesized at the Biochemical Core Facility in Emory University.

### Preparation and characterization of M2 VLPs

A full length M2 cDNA was generated by RT-PCR from total RNA isolated from MDCK cells infected with A/WSN/33 influenza virus (H1N1) and cloned into the pFastBac vector plasmid which was subsequently used to make recombinant Bacmid baculovirus DNAs using DH10Bac competent cells (rAcNPV, Invitrogen, Carlsbad, CA). A recombinant baculovirus (rBV) expressing influenza M2 protein was generated by transfection of sf9 insect cells according to the manufacturer's instruction. To produce influenza VLPs containing the wild type M2 protein (M2 VLPs), rBVs expressing M1 and M2 protein were co-infected into sf9 insect cells at multiplication of infection of 3. At 2 days post-infection, the infected cell culture supernatants were clarified by centrifugation (3000 rpm, 30 min) and then were concentrated by a QuixStand hollow fiber based ultrafiltration system (GE Healthcare, Piscataway, NJ). Influenza M2 VLPs were purified by sucrose gradient ultracentrifugation with layers of 20% and 60% (wt/vol) as previously described [26]. Influenza A virus M2 monoclonal antibody 14C2 (Abcam Inc., Cambridge, MA) was used for detection of M2 protein by western blotting of M2 VLPs. To quantify the amount of M2 incorporated into VLPs, His-tag affinity purified M2 protein produced by the rBV expression system was used as a standard. Western blots were analyzed by densitometer scanning using Alphaview software (Alpha Innotech, Santa Clara, CA). The morphology of VLPs was examined by electron microscopy at the Integrated Electron Microscopy Core Facility of Emory University as described [26].

### Immunization and challenge

For animal experiments, 6–8 weeks old female BALB/c mice (Harlan Laboratories, Indianapolis, IN) were immunized intranasally with 20 µg of M2 VLPs (M2VLP) or M1 only VLPs without M2 (Mock) with a single or two doses at 4 weeks interval. Four weeks after prime or boost immunization, mice were challenged with a lethal dose of A/PR/8/34 (3×50% mouse lethal dose (3 LD_50_) or A/Philippines/82 influenza virus (3 LD_50_). To determine the long-term protective efficacy, additional groups of mice (n = 9) were immunized intranasally with 10 or 20 µg of M2 VLPs two times (weeks 0 and 4) and challenged with a lethal dose (3 LD_50_) of A/PR/8/34 (H1N1) or A/Philippines/82 influenza virus 6 or 7 months post vaccination respectively. Mice were monitored daily to record weight changes and mortality (25% loss in body weight as the Institutional Animal Care and Use Committee (IACUC) endpoint). Full details of this study and all animal experiments presented in this manuscript were approved by the Emory University IACUC review board (approval number 179-2008 IACUC) and conducted under the guidelines of the Emory University IACUC. Emory IACUC operates under the federal Animal Welfare Law (administered by the USDA) and regulations of the Department of Health and Human Services.

### Determination of serum antibody responses specific to M2

Blood samples were collected before and at 3 weeks after each immunization and stored −20°C until analysis. M2 specific serum antibody responses were determined by ELISA using synthetic M2e peptide or inactivated purified virions (2 µg/ml) as a coating antigen as previously described [26], [49]. Briefly, HRP-conjugated goat anti-mouse IgG, IgG1 and IgG2a were used as secondary antibodies to determine total IgG and isotype antibodies. The substrate O-phenylenediamine (OPD) (Zymed, San Francisco, Calif.) in citrate-phosphate buffer (pH 5.0) containing 0.03% H_2_O_2_ (Sigma) was used to develop color. The optical density at 450 nm was read using an ELISA reader.

M2 expressing MDCK cells [50] were maintained in DMEM media with 7.5 µg/ml of puromycin (Invitrogen, Carlsbad, CA), 5 µM of amantadine (Sigma, St. Louis, MO) and 10% FBS (Invitrogen, Carlsbad, CA) at 37°C in air/CO_2_. Confluent M2 expressing MDCK monolayer cells were fixed by 0.05% glutaraldehyde or 10% buffered formalin (Sigma, St. Louis, MO) for 30 min at room temperature and used to determine antibody levels binding to M2 expressed on cell surfaces by ELISA as described [22]. M1 specific antibody responses were determined using the M1 protein peptide pool (2 µg/ml) derived from influenza A/New York/348/2003 (H1N1) virus (BEI resources, Manassas, VA).

### Nasal wash and BAL samples preparation

Nasal wash was collected by flushing through the trachea to nose with 500 µl of PBS for 5 individual mice and stored at −80°C until analysis. Bronchoalveolar lavage (BAL) fluid and cells were obtained by infusing 1 ml of PBS using a 25-gauge catheter into the lungs via the trachea. BAL cells were recovered and pooled from BAL fluid by centrifugation (1200 rpm, 5min) for the determination of cytokine secretion.

### Cross protective efficacy test of immune sera and effects of clodronate-liposomes

To test cross protective efficacy of immune sera *in vivo*, serum samples from immunized and mock control mice were pre-incubated with a lethal dose of influenza virus at room temperature for 30min as described [25]. A mixture of a lethal infectious dose of A/Philippines/82 (H3N2) or A/California/4/2009 (H1N1) influenza virus (3 LD_50_) and sera was administered to naive mice (n = 4 BALB/c), and body weight changes and survival rates were monitored daily. Liposome-encapsulated clodronate and control liposomes containing PBS only were prepared as previously described [51]. Four hrs prior to infection with virus- serum mixture, some groups of naïve mice (n = 6 BALB/c) were intranasally treated with clodronate-liposomes to deplete dendritic and macrophage cells as described [28], [29], [52]. Clodronate was a kind gift of Roche Diagnostics GmbH, Mannheim, Germany.

### Flow cytometry

The depletion efficacy of lung macrophage and DC cells was determined by flow cytometry as described [29]. Briefly, the homogenized lung tissues were incubated with DNase I (100 ug per ml, Sigma) and type IV collagenase (2 mg/ml, Worthington) for 30 min at 37°C, and then passed through a cell strainer (40 µm, BD, Franklin Lakes, NJ). The single-cell suspensions were stained with fluorescence conjugated antibodies specific to cell phenotypes (CD11c, CD11b). Lung macrophage and DC cells were gated according to their sizes and granularity defined in the forward light scatter (FSC) and side light scatter (SSC) plot and sorted based on their CD11b/CD11c profiles. Cell acquisition was performed with a dual-laser flow cytometer (LSR-II, BD Biosciences, Mountain View, CA ) and the data were analyzed using FlowJo software (Tree Star, INC., Ashland, OR).

### Lung viral titers and immune responses

Lung tissues were isolated from mice (n = 4) sacrificed at day 4 post challenge with influenza A/Philippines/82 H3N2 virus after 4 weeks after boost vaccination. Lung extracts were prepared using a mechanical tissue grinder with 1ml of PBS per each lung and viral titers were determined using plaque assay in MDCK cells as previously described [25], [26].

### Determination of T cell responses

Spleens were isolated from the same mice sacrificed at 4 day post challenge and single cell suspensions were prepared as described [53]. Interferon (IFN)- γ and interleukin (IL)-4 secreting cell spots were determined on Multi-screen 96 well plates (Millipore, Billerica, MA) coated with cytokine specific capture antibodies as described [53]. Briefly, 0.5×10^6^ spleen cells per well were cultured with or without M2e peptide (10 µg/ml) as an antigenic stimulator. After 36 h incubation, the number of IFN-γ or IL-4 secreting T cells was counted using an ImmunoSpot ELISpot reader (Cellular Technology, Shaker Heights, OH.).

### Statistical analysis

To determine the statistical significance, a two-tailed Student's t-test was used when comparing two different conditions. A *p* value less than 0.05 was considered to be significant.
